# A Large-Scale Dataset of Three-Dimensional Solar Magnetic Fields Extrapolated by Nonlinear Force-Free Method

**DOI:** 10.1038/s41597-023-02091-5

**Published:** 2023-03-30

**Authors:** Zhongrui Zhao, Long Xu, Xiaoshuai Zhu, Xinze Zhang, Sixuan Liu, Xin Huang, Zhixiang Ren, Yonghong Tian

**Affiliations:** 1 State Key Laboratory of Space Weather, National Space Science Center, Chinese Academy of Sciences, Beijing, 100190 China; 2 School of Astronomy and Space Science, University of Chinese Academy of Sciences, Beijing, 100049 China; 3 Peng Cheng Laboratory, Shenzhen, 518000 China; 4 School of Electronic and Computer Engineering, Peking University Shenzhen Graduate School, Shenzhen, 518055 China

**Keywords:** Astrophysical magnetic fields, Solar physics

## Abstract

It has been widely accepted that solar magnetic field manipulates all solar activities, especially violent solar bursts in solar corona. Thus, it is extremely important to reconstruct three-dimentional (3D) magnetic field of solar corona from really observed photospheric magnetogram. In this paper, a large-scale dataset of 3D solar magnetic fields of active regions is built by using the nonlinear force-free magnetic field (NLFFF) extrapolation from vector magnetograms of Helioseismic and Magnetic Imager (HMI) on Solar Dynamics Observatory (SDO). In this dataset, all space-weather HMI active region patches (SHARPs) with the corresponding serial numbers of national oceanic and atmospheric administration (NOAA) are included. They are downloaded from the SHARP 720 s series of JSOC every 96 minutes. In addition, each sample is labelled with a finer grained label for solar flare forecast. This paper is with the purpose of open availability of data resource and source code to the peers for refraining from repeated labor of data preparation. Meanwhile, with such a large-scale, high spatio-temporal resolution and high quality scientific data, we anticipate a wide attention and interest from artificial intelligence (AI) and computer vision communities, for exploring AI for astronomy over such a large-scale dataset.

## Background & Summary

The corona is the outermost atmosphere of the Sun and the origin of many solar eruptive activities. The measurements of coronal magnetic fields are critical to the study of the origin of solar activities, coronal heating, and other major scientific questions^1^. Up to now, there is only accurate measurement of photospheric magnetic field. The commonly used methods for measuring coronal magnetic field include polarization of coronal forbidden lines, radio observation, and coronal seismology. They all have their own notable shortcomings: (1) linear polarization of coronal forbidden lines only provides information of magnetic field direction^2,3^, and intensity information needs to be obtained by measuring circular polarization additionally. However, since circularly polarized signals are very weak and require a long exposure time, it is impossible to study the evolution of magnetic field in a short time; (2) radio observation can diagnose coronal magnetic field through measuring the influence of magnetic field on radio transmission or radio emission mechanism. However, radio emission mechanism is so complex that it is difficult to distinguish. Therefore, high-resolution spectral imaging observation is required for radio observation^1,4^; (3) the best result we can get from coronal seismology so far is the component of magnetic field perpendicular to the line of sight^5–10^. The newly developed method using coronal Fe X extreme ultraviolet spectroscopy can only measure magnetic field intensity, it is difficult to get magnetic field direction^11–15^. Therefore, coronal magnetic field measurement is still a big challenge although three-dimensional (3D) coronal magnetic field is extremely crucial for studying solar activities, such as solar flare and coronal mass ejection (CME). The 3D coronal magnetic field information was usually derived from numerical algorithms, such as magnetic field extrapolation from photospheric magnetogram^16–18^ and forward simulation^19–21^.

In this work, Wiegelmann’s Nonlinear Force-Free Field (NLFFF) extrapolation algorithm^22^ is employed to perform magnetic field extrapolation. The NLFFF is with the input of photospheric magnetogram and the ouput of 3D coronal magnetic field. The photospheric magnetogram is provided by the Space Weather HMI Active Region Patch(SHARP) which is extracted from SDO/HMI full-disk magnetogram according to the coordinates of active region (AR). In addition, the SHARP is remapped to the Lambert Cylindrical Equal-Area(CEA)^23^ coordinate system before extrapolation calculation. We aim to build the largest 3D magnetic field dataset with more than 73,000 samples, the size of which is far beyond the K. Kusano’s dataset^24^.

## Methods

### Introduction of the extrapolation method

The solar coronal magnetic field can be described approximately by the force-free field due to low plasma *β* = 2*μ*_0_*p*/*B*^2^ in the corona. The force-free field equations are given by

1 $$\left(\nabla \times {\bf{B}}\right)\times {\bf{B}}={\bf{0}}$$

2 $$\nabla \cdot {\bf{B}}=0$$

subject to the boundary condition

3 $${\bf{B}}={{\bf{B}}}_{obs}\quad {\rm{on}}\;{\rm{the}}\;{\rm{bottom}}\;{\rm{boundary,}}$$

where **B** is the three-dimensional (3D) magnetic field, **B**_*obs*_ is the real measured vector magnetic field in the photosphere.

In this work, we employ a three-level multiscale approach developed by Wiegelmann^22,25^ to reconstruct coronal magnetic field. In this method, coronal magnetic field is deduced by minimizing the following optimization function

4 $$L={\int }_{V}{w}_{f}\frac{| (\nabla \times {\bf{B}})\times {\bf{B}}| }{{B}^{2}}+{w}_{d}| \nabla \cdot {\bf{B}}{| }^{2}{d}^{3}V+\nu {\int }_{S}({\bf{B}}-{{\bf{B}}}_{obs})\cdot {\bf{W}}\cdot ({\bf{B}}-{{\bf{B}}}_{obs}){d}^{2}S$$

where *v* is a Lagrangian multiplier which controls the injection speed of the boundary conditions, and *w*_*f*_ and *w*_*d*_ are two weighting functions, **W** is a space-dependent diagonal matrix whose elements are inversely proportional to the estimated squared measurement error of the respective field component. The minimization of Eq. (4) is achieved by taking functional derivative of Eq. (4) with respect of the iteration parameter *t*. Note that if *L* = 0 is achieved, the force-free Eqs. (1) and (2) can be solved.

### Raw data selection from JSOC

**Fig. 1:**
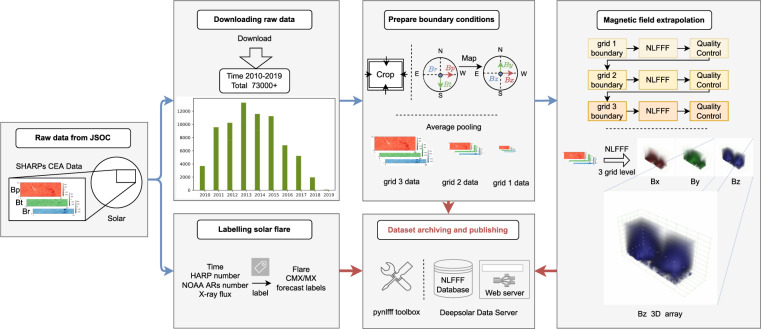
The overall process of NLFFF dataset construction.

As shown in Fig. 1, we start to build the dataset by selecting the SHARPs data^26^ published on Joint Science Operations Center (JSOC) website^27^, according with the condition that the center longitudes of the SHARPs are less than 30°. The detailed processing steps are as follows.

- First, we download data from the SHARP 720 s series of JSOC^26^ (including “Bp.fits”, “Bt.fits” and “Br.fits”) every 96 minutes from 2010 to 2019;
- Second, we pick out the SHARPs whose HMI Active Region Patch (HAPR) serial numbers have at least one corresponding NOAA AR serial number;
- Third, maximum Stonyhurst longitude (LON_MAX) and minimum Stonyhurst longitude (LON_MIN) of an SHARP should satisfy with |*LON*_*MAX* + *LON*_*MIN*|/2 < 30°.

We download the SHARP data^26^ with a SunPy^28^ affiliated package called “drms”^29,30^. We use “drms” to filter data to be downloaded by setting the cadence, LON_MAX and LON_MIN parameters. Parsing the header of the raw fits file, we can obtain the correspondences between HARP serial number and NOAA serial number, which also can be accessed via (http://jsoc.stanford.edu/doc/data/hmi/harpnum_to_noaa/).

### Preparing boundary conditions

To allow a batch processing, we develop a Python code with fixed configuration to prepare boundary conditions. This process consists of two steps: First, determining the parameters of Eq. (4); Second, generating data and files according to the parameters. To solve Eq. (4), the parameters we need to determine beforehand are **B**_*obs*_, ***ν***, **W**, *w*_*f*_, *w*_*d*_:

- Parameter **B**_*obs*_, **B**_*obs*_ represents the real observation of photospheric magnetic field. Related to **B**_*obs*_, the pixel size of a magnetogram in the *x*-*y* plane, *nx* and *ny*, might be revised to be a multiple of 4 to fit the three-level multigrid method. The pixel size *nz* in the *z* axis is set to 3/8*(*nx* + *ny*) and rounds up to a multiple of 4.
- Parameter *v*, *v* controls the injection speed of the boundary conditions. In this work, the vector magnetogram is slowly injected with *v* = 0.001 in each extrapolation. And we do not employ the pre-processing before magnetic field calculation as the majority of HMI vector magnetograms are close to the force-free state.
- For Parameter **W**, we use *B*_*T*_/*max*(*B*_*T*_) to generate mask **W**, where ${B}_{T}=\sqrt{b3d{x}^{2}+b3d{y}^{2}}$ is the strength of the transverse magnetic field.
- Parameters *w*_*f*_ and *w*_*d*_ are weighting functions which usually equal to 1 in the region of interest (inner (*nx*-*nd*)×(*ny*-*nd*)×(*nz*-*nd*) physical box), and drop to 0 in a *nd*-pixel boundary layer toward top and lateral boundaries of the full *nx*×*ny*×*nz* computational domain. Note that Wiegelmann’s NLFFF extrapolation method sets top and side boundary conditions to the value of the potential field which may not be consistent with the force-free condition. Thus, the *nd*-pixel boundary layer improves the results in the physical box by reducing influence from top and side boundaries. However, SHARP uses automated method to cut out active regions, which may produce active regions close to the boundaries. To these active regions, setting *nd* > 0 is likely to exclude parts of them from physical box. Therefore, we set the size of boundary layer *nd* = 0 in the computations. It is worth noting that Wheatland’s optimization method^31^ without the buffer zone has also been applied in many coronal magnetic field reconstructions^32–34^.

**Fig. 2:**
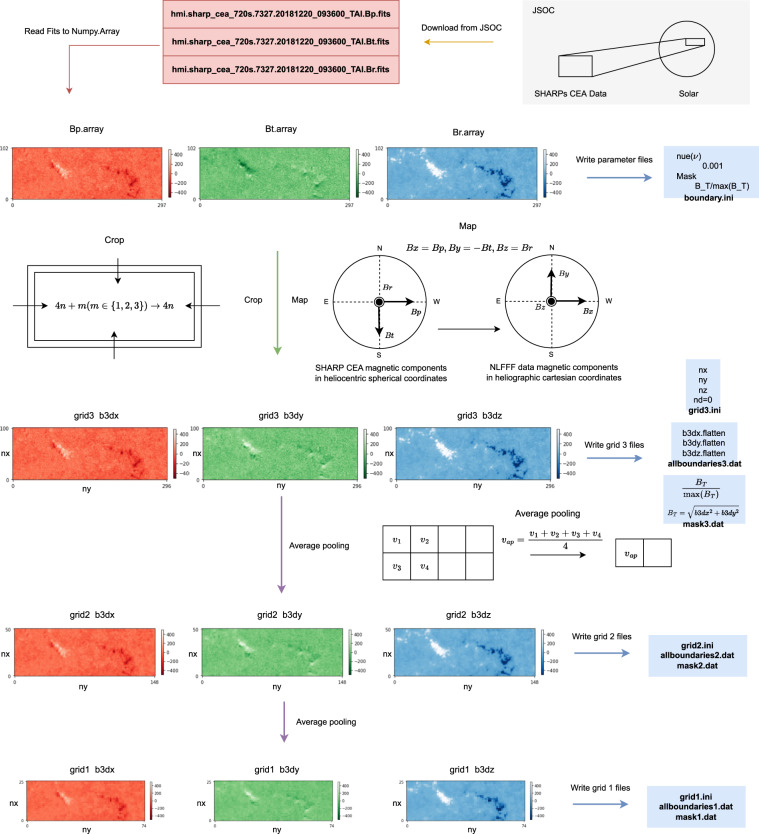
The process for preparing boundary conditions.

Once the parameters(*nx*, *ny*, *nz*, *v* = 0.001, *w*_*f*_ = 1, *w*_*d*_ = 1, *nd* = 0) were determined, we can generate boundary data files, including “allboundaries1/2/3.dat”, “grid1/2/3.ini”, “mask1/2/3.dat” and “boundary.ini”, which will be used in the NLFFF extrapolation. As shown in Fig. 2, the process of preparing boundary conditions can be divided into data reading, cropping and mapping; generating parameter files and boundary files which includes three different resolutions for the purpose of multiscale optimization^22^. The detailed processing can be divided into 5 steps:

- Step 1: Generate the **B**_*obs*_ file, first read Bp, Bt and Br from “hmi.sharp_cea_720s.X.Bp.fits”, “hmi.sharp_cea_720s.X.Bt.fits” and “hmi.sharp_cea_720s.X.Br.fits”; second crop *nx*, *ny* of Bp, Bt, Br to a multiple of 4; third let “b3dx = Bp”, “b3dy = -Bt” and “b3dz = Br”, where “b3dx”, “b3dy” and “b3dz” are the three components of vector magnetic field in Cartesian coordinate for extrapolation; finally, “b3dx”, “b3dy” and “b3dz” are flatten in column-major (Fortran-style) and written to “allboundaries3.dat”.
- Step 2: Generate parameter file, i.e. writing “boundary.ini” which records *v* as “nue 0.001” and mask as “Mask B_T/max(B_T)”, writing “grid3.ini” file which records *nx*, *ny*, *nz*, and *nd* = 0 (0 pixel boundary layer).
- Step 3: Generate “mask3.dat” which record mask ${\bf{W}}=\frac{{B}_{T}}{max\left({B}_{T}\right)}$ at each pixel in the magnetogram.
- Step 4: Downsample “b3dx”, “b3dy”, and “b3dz”, and repeat Steps 1–3 to generate three files “allboundaries2.dat”, “grid2.ini” and “mask2.dat” for the extrapolation at the second grid level.
- Step 5: Repeat Step 4 to generate “allboundaries1.dat”, “grid1.ini” and “mask1.dat” for the extrapolation at the third (the coarsest) grid level.

### Calculation process

**Fig. 3:**
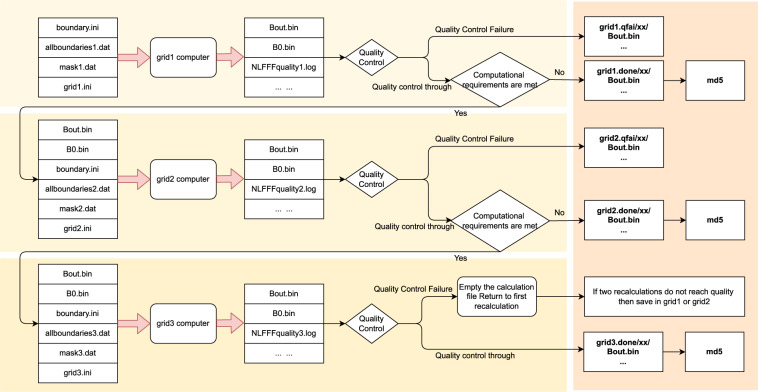
Process of batch calculation of magnetic field extrapolation.

After preparing the boundary condition, the Wiegelmann’s NLFFF method^22^ is employed to solve the force-free equation to calculate coronal magnetic field. For batch processing, the Python code is developed to handle the bulk data. For the best use of computing resource, we split the three grid levels of computation to make the best use of computing resources when binding tasks to the CPU core. As shown in Fig. 3, the calculation process is divided into three levels correspond to three grid levels. Each level consists of three steps: magnetic field calculation, quality control and calculation verification. Note that level 2 calculation depends on the result of level 1, and level 3 calculation depends on the result of level 2. To accelerate computing, we use multiple processes with each computing process binded to a CPU core. Memory and the number of CPU cores are allocated according to the task’s complexity. We found that the matching of CPU core *cpu*_*core* = (*nx*∗*ny*∗*nz*)/(1024∗1024∗20) + 1 and memory *memory* = *cpu*_*core*∗4GB is a good choice. In addition, a single process for smaller magnetograms and multiple processes for larger magnetograms can maximize the use of computational resources. After magnetic field calculation, the quality check is performed on the output of the calculation, where the angle between magnetic field and electric current less than 30° would pass the quality check, i.e., ∠(*B, J*)≤30° which is recorded in “NLFFFquality.log”. If the quality check does not pass, we would calculate once more to exclude the failures due to hardware or system failure, such as hard disk error, memory error, and network error, etc. If the calculation still does not pass, we would save the corresponding level file and label it as quality failure, namely “qfail”. Once the quality check passes, we will judge whether the additional computing resources are allowed for the next level calculation. The computing resource mainly refers to the size of computer memory and the number of CPU cores. If the computing resource meets the need of next level calculation, the next level calculation will continue. Otherwise, the result is saved and labeled as “done”, and finally the Message-Digest Algorithm (MD5) hash of “Bout.bin” is calculated for verifying file integrity after transmission.

### Flare labeling

For solar flare forecast, we also label the samples in the dataset with “non-flare” and “flare”(denoted by “0”, “1”). In addition, it is also worth noting that there are very few “flare now” samples which are not used for solar flare forecast modeling, but may be useful for other studies. We give this kind of samples the label “2”. “non-flare” means there are no flares within a given time period in the future (e.g., 24 hours), “flare” means there is at least a flare within the given time period, “flare now” means there is a ongoing flare currently. In addition, the amount of flare level variation within a certain time period in the future is labelled. We use the NOAA released flare list, which was recorded by Geostationary Operational Environmental Satellite (GEOS), as the baseline for labeling. Compared to the previous labeling method^35^, we provide a finer grained label in this study for possible more applications in the future besides flare forecast. The label information (as listed in Table 3) includes current flare information, maximum flare information in the next 6, 12, 24 and 48 hours, with/without “CMX” or “MX” level flares (“X” is the most violent, “M” is the next, and “C” is the least) in 24 and 48 hours, and maximum level change in 24 and 48 hours.

**Fig. 4:**
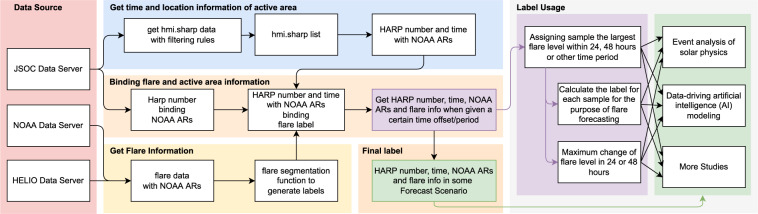
Flare labeling process.

The labeling process is shown in Fig. 4. First, we get the HARP number with time from the filename of a FITS file. For example, “hmi.sharp_cea_720s.4201.20140607_013600_TAI.Bp.fits” indicates the HARP number is 4201, the temps atomique International (TAI) time is 2014/06/07 01:36:00.

Second, we download x-rays flare data from National Centers form Environmental Information (NCEI)^36^, Space Weather Prediction Center (SWPC)^37^ and Heliophysics Integrated Observatory (HELIO)^38^. After de-duplicating, cleaning and proofreading, we establish the relationship between flare level and NOAA number, forecasting period.

Then, we can establish a simple formula with the inputs of NOAA number and forecasting period, and with the output of the corresponding flare information, including maximum flare level and flare identity within a given forecasting period (0 hours, 6 hours, 12 hours, 24 hours, 48 hours).

Third, we can visit (http://jsoc.stanford.edu/doc/data/hmi/harpnum_to_noaa/) to get the correspondences between SHARPs and NOAA ARs. If a HARP corresponds to more than one NOAA ARs, the final flare level of this HARP is the largest flare level of the associated NOAA ARs. Then, we can establish a connection between “harp_time” and flare information. Thus, for each sample in the dataset, we can judge whether there is a flare and relevant flare information given a certain time period. We have already labeled all of the samples in the dataset, assigning them the largest flare level within 24, 48 or other time period. Then, we can easily deduce the labels for all flare forecast modes to each sample. As shown in Table 1, the first column gives several forecast modes, where the three labels “0”, “1” and “2” represent “no flare”, “flare” and “flare now” for each forecast mode, respectively. From Table 1, each row lists the conditions that the corresponding forecast mode should meet. In addition, subtracting the current flare level from the maximum flare level in the future 48 hours can tell us the maximum change of flare level in 48 hours. These labels can be used in both event analysis of solar physics and data-driving artificial intelligence (AI) modeling.

**Table 1:** The description of flare forecast mode (include “CMX” and “MX” flares in 24 hours and 48 hours respectively listed in the first column; each row lists the conditions that a forecast model should meet).

Flare forecast mode		Flare now		Flare in future 24 hours		Flare in future 48 hours	
		MX-level	CMX-level	MX-level	CMX-level	MX-level	CMX-level
MX flare in 24 hours	0						
	1			✓			
	2	✓					
CMX flare in 24 hours	0						
	1				✓		
	2		✓				
MX flare in 48 hours	0						
	1					✓	
	2	✓					
CMX flare in 48 hours	0						
	1						✓
	2		✓				

### Database archiving and publishing

As shown in Fig. 3, after getting the output and log files generated by “grid1.done”, “grid1.qfail”, “grid2.done”, “grid2.qfail”, “grid3.done”, the MD5 hash is calculated for the output file “Bout.bin” to verify its integrity. Here, “grid*n*” implies that the NLFFF computing reaches the maximum stage of *n*, “done” and “qfail’ indicate the success and failure of NLFFF computing respectively. The NLFFF succeeds if the angle between magnetic field *B* and current *J*, namely ∠(*B, J*), is less than 30°, otherwise the NLFFF would fail to pass the quality check. For example, “grid3.done” means that the NLFFF reaches the maximum stage of 3, and the computing result is satisfied, namely ∠(*B, J*)≤30°. For explaining these symbols more clearly, they are compared in Table 2 with respect to the computing level and the quality check. Then, we parse the header of the original SHARP fits file to get the HARP number and time for flare labeling according to the flare records published by NOAA.

**Table 2:** Description of product quality.

	Conditions	grid1.qfail	grid1.done	grid2.qfail	grid2.done	grid3.done
**Level 1**	Calculated to level 1	✓	✓	✓	✓	✓
	Level 1 quality pass, is ∠(*B*, *J*) in NLFFFquality1.log is less than 30°		✓	✓	✓	✓
**Level 2**	Calculated to level 2			✓	✓	✓
	Level 2 quality pass, ∠(*B*, *J*) in NLFFFquality2.log is less than 30°				✓	✓
**Level 3**	Calculated to level 3					✓
	Level 3 quality pass, ∠(*B*, *J*) in NLFFFquality3.log is less than 30°					✓

**Fig. 5:**
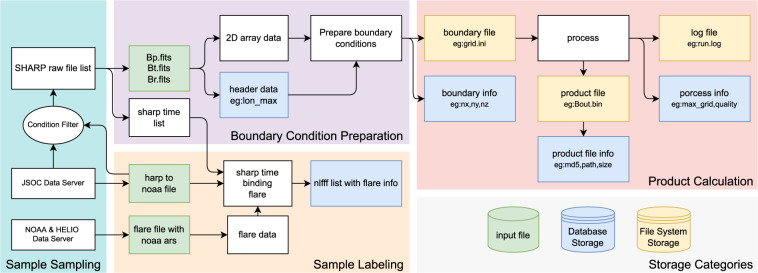
Archiving and publishing.

Then, the storage path, MD5 hash, storage space, header of original fits file and flare label for each computed coronal magnetic field are written into the database, publishing over the web page. The whole process is shown in Fig. 5.

## Data Records

### Description of data records

As shown in Figs. 5, 7, the process of building dataset is divided into two parts: NLFFF computing and labeling, each of which consists of four major steps: downloading raw data, preparing, processing and archiving. According to the data property, there are two types of storage, namely database storage and file system storage, distributed in 3 locations, “nlfff_file”, “label_file”, and “archive_db”.

All dataset files and the Python source code are publicly available. They can be downloaded from the project website^39^. At the same time, “nlfff_file” summary information, “label_file” and “archive_db” can be obtained from the figshare collection^40^.

**Fig. 6:**
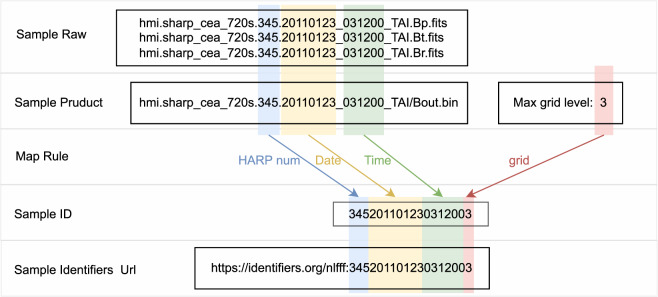
Sample identifiers.

For “nlfff_file”, the figshare collection^40^ provides summary information (as shown in rows “raw”, “prepare”, “process” and “archive” of Table 3) for each sample in “csv” format. This part is divided into original data and product data:

- Original data is SHARP CEA raw fits header. The full fits file, such as “hmi.sharp_cea_720s.X.Bp/Bt/Br.fits”, can be downloaded from the official raw data release webpage^27^.
- Product data is NLFFF product sample information (as shown in rows “prepare”, “process” and “archive” of Table 3) for each sample. The complete file list for each sample is shown in Table 4, which can be accessed through the project website^39^, and can also be accessed through Identifiers.org^41^. As shown in Fig. 6, where a sample^42^ can be acquired by the HARP number, time and max calculation grid level.

**Table 4:** NLFFF data file list.

Generation stage	File name	Description
prepare	grid1.ini, grid2.ini, grid3.ini	Information about the grid used for the corresponding level
	mask1.dat, mask2.dat, mask3.dat	The mask data used for the corresponding level
	allboundaries1.dat, allboundaries2.dat, allboundaries3.dat	The boundaries data used for the corresponding level
	boundary.ini	Boundary and algorithm information
process	Bout.bin	Nonlinear force-free field
	B0.bin	Potential field, due to storage problems, this part of the data is partially saved
	NLFFFquality1.log, NLFFFquality2.log, NLFFFquality3.log	Corresponding level of product quality
	prot1.log, prot2.log, prot3.log	Corresponding level iteration log information
	step1.log, step2.log, step3.log	Information on the number of iterative steps for the corresponding level
	Energy.log	Run Energy Log,If you only run to grid1, this part may not have
archive	run.log	The detail of run log

**Fig. 7:**
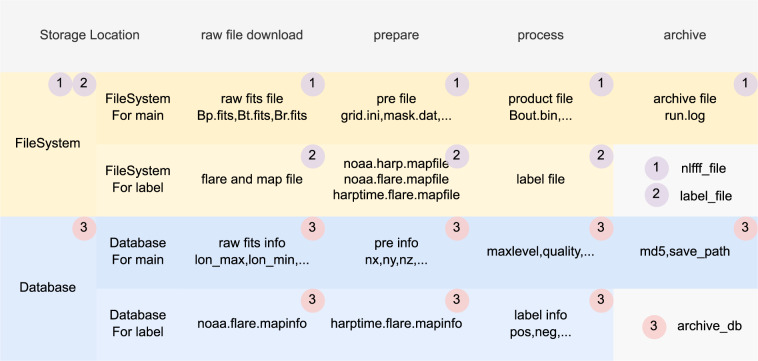
File recording and storage.

**Table 3:** NLFFF data.

	Name	Type	Description
raw	harpnum_trec	timestamp	HARP number and time from raw fits name
	sync with raw fits	sync raw	find from http://jsoc.stanford.edu/ajax/lookdata.html?ds=hmi.sharp_cea_720s
prepare	grid_x	int4	We finally can calculate the *nx*, *ny*, *nz* corresponding to the level, that is, the *nx*, *ny*, *nz* corresponding to the saved Bout.bin file
	grid_y	int4	
	grid_z	int4	
process	bout_maxlevel	int4	The final calculated level,the level of the last saved bout
	bout_quality_value	float8	Set to True if ∠(*B*, *J*) in quality check is less than 30°, else False.
	bout_quality	bool	The final calculated quality,the quality of the last saved bout
archive	bout_path	text	The path where Bout is saved, and other files in the same subdirectory as Bout
	bout_md5	varchar(32)	The MD5 hash of Bout
	bout_size	int8	The size of Bout
	identifiers	int8	The identifiers of sample
	batch	int8	Calculated batches, other calculated batches may be available in the future
flare label	now_flare_level	int4	Current flare level
	now_flare_id	int4	Current flare id
	h6_flare_level, h12_flare_level, h24_flare_level, h48_flare_level	int4	Maximum flare levels in 6, 12, 24 and 48 hours, respectively
	h6_flare_id, h12_flare_id, h24_flare_id, h48_flare_id	int4	The ids corresponding to the maximum flare levels in 6,12,24,48 hours, respectively
	h24_posmx	int8	0 - non-flare sample, No MX or CMX level flares in the future 24 or 48 hours; 1 - flare sample - MX or CMX level flares in the future 24 or 48 hours; 2 - flare now sample, MX or CMX flares now
	h24_poscmx	int8	
	h48_posmx	int8	
	h48_poscmx	int8	
	h24_delta05	int8	Maximum change in grade in future 24 hours
	h48_delta05	int8	Maximum change in grade in future 48 hours

For “label_file”, the figshare collection^40^ provides the files as shown in Table 5. The sorted flare data information is shown in Table 6, and the label data is shown in row “flare label” of Table 3. The raw file can be downloaded from the official release webpage^36–38^.

**Table 5:** Flare information file list.

Generation stage	File name	Description
raw_file	flare_raw	The folder containing the original flare information for download
	all_harps_with_noaa_ars.txt	Mapping update for HARP number and NOAA number
prepare	knoaa_vflaretimelist.pickle	The dictionary with key NOAA number, value flare time list is saved as python pickle
	ksharp_vnoaa.pickle	Key is HARP number,value is NOAA number list of dictionaries saved as python pickle
process	label.csv	Sample Label Information

**Table 6:** Flare data.

Name	Type	Description
deeps_flare_id	int4	The id that uniquely identifies the flare information in the deepsolar database system
start_datetime	timestamp	Flare start time
peak_datetime	timestamp	Flare end time
end_datetime	timestamp	Flare peaking time
xray_class	varchar(1)	Flare level class
xray_intensity	int4	Intensity of raw data multiplied by 10
latitude	int4	latitude
longtitude	int4	longtitude
noaa_ar	int4	Corresponding NOAA active region number
source	varchar(16)	Data source

The “archive_db” is a table of NLFFF product and flare information in “SQLite” database format. It stores the information both Tables 3, 6. This database can be accessed via the figshare^40^ and the website^39^. Users can retrieve the data they are interested in by imposing query condition on the dataset through database “archive_db”. For the users who need a large amount of data and do not wish to download 200 TB from the platform^39^, we recommend contacting us via the website^39^ to arrange physical transfer, such as via mailed hard disks.

### NLFFF Data format

**Fig. 8:**
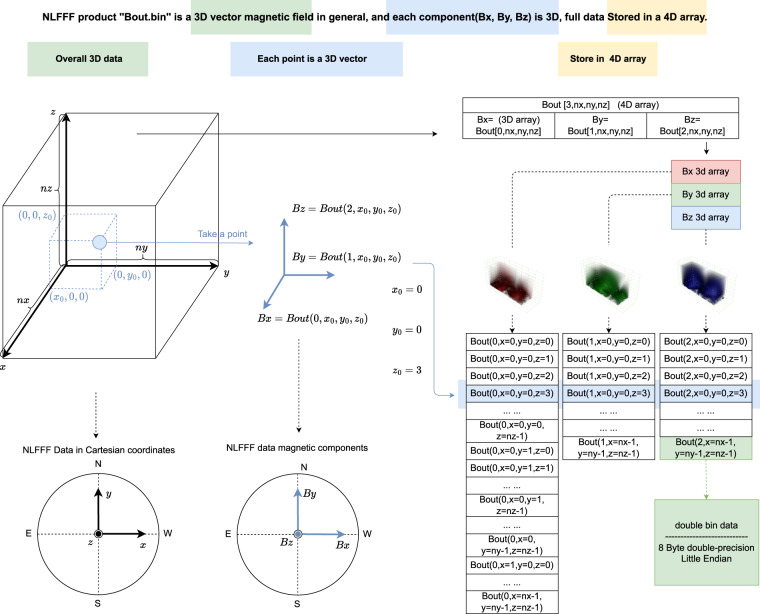
Description of NLFFF product “Bout.bin”.

The computed NLFFF data is stored in the “Bout.bin” files, each of which contains three-dimensional vector magnetic field in Cartesian coordinates. In Cartesian coordinates, each point contains three magnetic field components, “Bx”,“By” and “Bz”, in Gaussian units, as shown in Fig. 8. A “Bout.bin” file can be regarded as a four-dimensional array with the size of (3, *nx*, *ny*, *nz*), where the first dimension indicates which of the three magnetic field components, and the other three dimensions give the size of magnetic field “Bx/y/z”. The three components, “Bx”, “By” and “Bz”, are stored in sequence in a binary file, with row-major (C-style) order and 8-byte double precision little endian.

It is worth noting that the *nx*, *ny*, *nz* of “Bout.bin” and the corresponding “grid.ini” should be consistent. As mentioned above, there are maximum three levels of nlfff calculation for the collected ARs. After each level of calculation, there is a quality checking process to decide whether to proceed with the next calculation. From our rough count, most of the ARs are calculated to the third level, namly “grid3”, but there are still a small number of ARs calculated to the second or the first levels, namely “grid2” or “grid1”. For most of the collected samples in the database, the computed NLFFF data has almost the same spatial resolution along longitude and latitude as the sharp cea raw data, but there may still exist a small difference of 0–3 pixels since the input magnetogram was cropped to the resolution of multiples of 4 in the previous steps. This pixel-level difference can be ignored in our task. However, it may need to be considered in the tasks where pixel-level discrepancy is highly concerned. In addition, the *z*-direction and *x/y*-direction are equally proportional, which means that the actual height can also be inferred from the latitude and longitude.

## Technical Validation

To ensure the quality of the final product, quality control is performed after each level extrapolation computing, the details of which can be found in Fig. 3. If the quality control is not satisfied, the calculation is performed again from level 1. This ensures that the data is reliable every time as it goes to the next level.

**Fig. 9:**
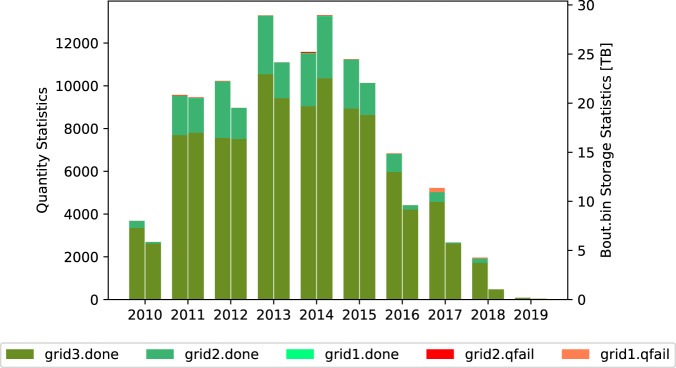
Statistics of product quantity and storage. In “grid*n*.done” or “grid*n*.qfail”, “done” means ∠(*B*, *J*)≤30°, “qfail” means ∠(*B*, *J*) > 30°, *n* represents the maximum level of extrapolation computing.

**Fig. 10:**
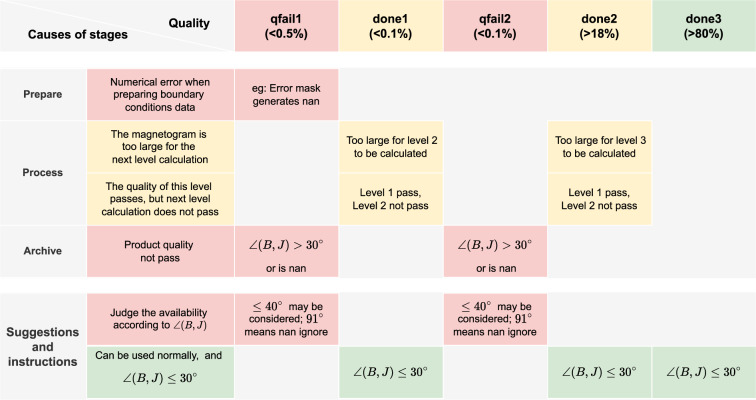
Description of product. In “grid*n*.done” or “grid*n*.qfail”, “done” means ∠(*B*, *J*)≤30°, “qfail” means ∠(*B*, *J*) > 30°, *n* represents the maximum level of extrapolation computing.

In Fig. 9, we present the distribution of samples in chronological order, and the corresponding storage space of “Bout.bin”. In Fig. 10, we describe product quality as well as instructions and recommendations for use. We can observe that the samples in 2013 and 2014 account for a large portion since these two years are the solar maximum year of the solar cycle 24. In addition, Table 7 lists the statistics of the number/percent of samples for each situation. It can be observed that ‘grid3.done” accounted for 80% which means that most of the samples are calculated up to the third level. The “grid2.done” accounted for 18%, indicating 18% samples are calculated up to the second level. It can be also noticed that the large number of samples of “grid2.done” appear in 2011–2015. There are two cases for “grid2.done”: (1) the input magnetogram is too large, resulting in computing resource is run out at the second level; (2) although the computing of the second level succeeds, the quality of the third level calculation “grid3” is not satisfied. “grid1.done” accounts for a very small proportion, less than 0.1%. “grid1.done” means that the first level calculation “grid1” succeeds and the quality passes the checking, however the next level calculation fails due to limited computing resource possibly. “grid2.qfail” also accounts for less than 0.1%. “grid2.qfail” means that the calculation of grid2 succeeds but the quality does not pass the checking. “grid1.qfail” accounts for 0.48%, which means that the calculation of “grid1” fails or the quality does not pass the checking. The reason is twofold. First, the quality of the original data is not satisfied, e.g., the presence of the “Not a Number (NaN)” value in the original SHARPs data. Second, the computed result does not pass the quality checking.

**Table 7:** Statistics of product quantity.

	grid3.done	grid2.done	grid1.done	grid2.qfail	grid1.qfail	all
**Count**	59479	13805	50	60	353	73747
**Count_percent[%]**	80.65	18.72	0.07	0.08	0.48	100
**Bout size[TB]**	116.88	20.63	0.07	0.08	0.04	137.71
**Size_percent[%]**	84.88	14.98	0.05	0.06	0.03	100

In “grid*n*.done” or “grid*n*.qfail”, “done” indicates ∠(*B*, *J*)≤30°, “qfail” indicates ∠(*B*, *J*) > 30°, *n* represents the maximum level of extrapolation.

**Fig. 11:**
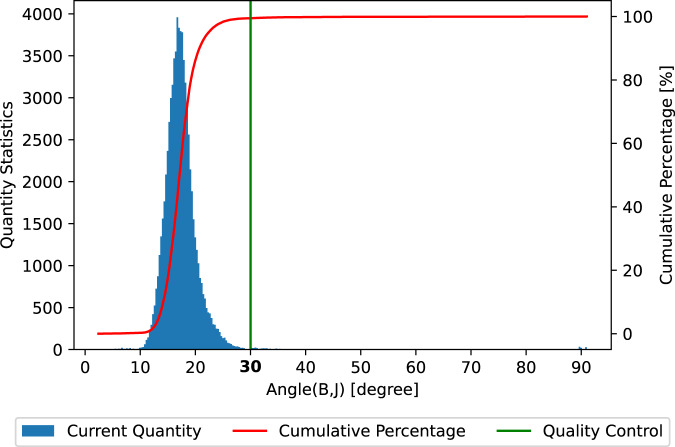
Distribution of product with respect to its quality measured by ∠(*B*, *J*).

In addition, we classify all calculated results according to the quality measurement mentioned above, namely ∠(*B*, *J*). Then, we count the proportion of each class of data, demonstrated in Fig. 11. It can be observed that 50% data has the ∠(*B*, *J*) below 17°, 90% data is below 20°, and 99% is below 30°, where the ∠(*B*, *J*) from 0 to 90 is taken from “NLFFFquality.log”, while the value of 91° represents a “NaN”.

## Usage Notes

In this work, a large repository of solar nonlinear force-free field 3D magnetic fields is built. In the repository, each item also includes several associated parameters for describing a 3D magnetic field and flare label. The resource of this repository aims to facilitate the research for probing the true coronal magnetic field evolution, uncovering topological structure and geometric structure of coronal magnetic field as solar bursts occur, and forecasting solar flares. In terms of volume, spatial resolution and temporal resolution, this repository is all far beyond the previous ones, such as the K. Kusano’s dataset^24^. With such advantages, we also expect this repository could be widely spread among the communities of artificial intelligence, computer vision and video/image processing to promote and validate their algorithms in real application scenarios.

### Physical properties of the magnetic field in the active area

We have collected almost all sharp cea files with the corresponding NOAA AR numbers from 2010 to 2019 and with temporal resolution of 96 minutes. For maintaining and updating this repository, we have developed batch processing code to collect the latest data and update the database.

### Flare forecast study

With higher temporal resolution and larger amounts of data, it is now possible for deep learning models to tap into deeper physical laws and solar flares precursors. More data means more perspectives can also be analyzed and compared.

### 3D point cloud dataset

The output of nlfff is also a 3D point cloud. Thus, a 3D point cloud dataset for scientific research is built. The data volume of the established repository has over 200TB, which implies a big challenge for data processing, compression, storage, feature extraction and computer vision tasks.

### Flexibility of the resource

By providing full size images same as the original file, we allow researchers to fine-tune object recognition and other computer vision algorithms without the constraints of only having the regions of interest. Using high-resolution images, researchers are able to down-sample the images freely and are able to test algorithms with a wide range of settings and parameters. By providing raw image parameters, nlfff product and flare information, we provide multiple levels of data that could facilitate researches in both traditional image retrieval, flare forecast, magnetic field evolution, and deep learning applications.

For the reproduction of the whole process, all python source code is publicly accessible via our website^39^. By releasing all documentation and code, the users can reproduce and update this database by themselves when they need. In addition, we are planning to provide online service of high-performance magnetic extrapolation computing for users who upload their own data of interest.

**Fig. 12:**
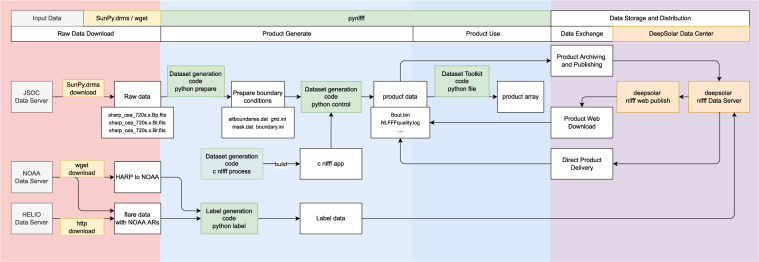
Code and usage flow.

## Data Availability

In order for this dataset to be fully reproducible and expandable in the future, we have open-sourced all the Python code used to generate and validate the resource in the following code repository (https://github.com/deepsolar/pynlfff) and can be downloaded directly via pip as pip install pynlfff. The code can be divided into three parts, dataset generation code, label generation code and dataset Toolkit code. The dataset generation code is for generating the dataset, label generation code is for labeling flare information to nlfff data list, and dataset Toolkit code is for manipulating the data. The whole process of code usage is shown in Fig. 12 to explain this usage more clearly. The tools and examples for getting original Bp, Bt and Br fits can be found at (https://github.com/mbobra/SHARPs). **Dataset generation code** The code of dataset generation mainly consists of three different components. The first component contains the preparing boundary conditions programs. This utility uses Bp.fits, Bt.fits and Br.fits of “hmi.sharp_cea_720s” to generate “boundary.ini”, “mask.dat”, “grid.ini” and “allboundaries.dat” for the next step. This code is multi-threaded for computing efficiency, allowing the users set the number of threads. Note that if the raw data file is corrupted or with the quality problem, the boundary conditions file may not be generated properly. The corrupted raw files may report an error when operating them, e.g., the quality problem of raw data may cause generated “mask.dat” file with “NaN”. The second component is for magnetic field calculation, consisting of Python code for computing flow control and magnetic field extrapolation module provided by Wiegelmann’s team^22^. The Python code is responsible for scheduling and controlling core computing, specifying the number of running processes, binding tasks and cores, adaptively assigning cores according to the task, maximizing the use of computing resources, quality control, and logging, etc. The third component is magnetic field calculation written by C language program. It is not included in our published “pynlfff” package since its copyright is owned by Wiegelmann’s team^22^. The C code needs to be compiled beforehand, and “pynlfff” provides bash scripts to automatically compile and generate single-process and multi-process programs. In addition, we have rewritten multi-grid bash scripts to perform magnetic field extrapolation for each layer separately. Python and C should be implemented together, using single-process C programs for small tasks and multi-process C programs for large tasks. In addition, we allocate computing cores according to the task size and employ core binding technique to maximize the use of computing resources. **Dataset Toolkit Code** After getting the dataset file, you can implement your own program to read the product file “Bout.bin” based on the storage structure of the product file “Bout.bin” which has been described in subsection NLFFF Data Format, and we provide a toolkit for python implementation to help you with the reading operation. **Flare label generation code** As shown in Fig. 4, pynlfff already implements these processes and has updated the label information in the project website^39^, if there is any other information that needs to be customized, it can be done through pynlfff or by modifying the pynlfff code.
